# A Commentary on the Potential Use of Oral Microbiome in Prediction, Diagnosis or Prognostics of a Distant Pathology

**DOI:** 10.3390/dj10090156

**Published:** 2022-08-24

**Authors:** Egija Zaura

**Affiliations:** Department of Preventive Dentistry, Academic Centre for Dentistry Amsterdam, Vrije Universiteit Amsterdam and University of Amsterdam, Gustav Mahlerlaan 3004, 1081 LA Amsterdam, The Netherlands; e.zaura@acta.nl

**Keywords:** oral microbiome, oral health, general health, prediction, biomarkers, oral ecosystem

## Abstract

In health, the oral microbiome is in balance with its host. If this balance is lost, this symbiosis is replaced by dysbiotic microbial communities, which are thought to affect the rest of the body either directly or via metabolites or pro-inflammatory molecules. The association of oral microbiome with general health has led to attempts to use oral microbial biomarkers for the prediction, diagnosis or prognosis of distant pathologies such as colorectal carcinoma or pancreatic cancer. These attempts however have no chance to succeed if the complexity of the oral ecosystem and the interplay of environmental, behavioral and biological factors is not taken into account. Standardized, well-documented oral sample collection procedures together with detailed clinical oral examination and behavioral data are the prerequisites for the successful evaluation of the oral microbiome as a potential biomarker for distant pathologies.

## 1. Introduction

Traditional medicine, especially in Asian countries, relies on visual inspection of the patient’s tongue for assisting diagnosis of systemic conditions. Oral health is believed to mirror one’s general health. As early as 400 years BC, the father of medicine, Hippocrates, observed an association between the extraction of a decayed tooth and resolution of arthritis. In the modern medical world, though, oral and general health are addressed as separate entities by oral and medical professionals, respectively. However, the link between the oral and systemic health cannot be denied: among others, associations have been found between poor oral health and increased risk for cardiovascular disease, metabolic syndrome, diabetes, pregnancy complications, respiratory diseases, rheumatic diseases, cancers and even Alzheimer’s disease [1,2,3,4].

If we look holistically at the human body, interconnectivity of different body parts seems only logical. A truly holistic view though requires acknowledgement of our invisible fellow inhabitants together with the full repertoire of their functions—the microbiome [5]. Evolution of life on Earth started with its oldest form—anaerobic bacterium. This ancient bacterium can be seen as a common ancestor to all other forms of life, including humans: 37% of our genes stem from bacteria [6]. The ubiquitous presence of microbial life has led to natural and symbiotic relationships between microbes and all other forms of life, including humans. In fact, the human genome is quite limited and microbial genes are mandatory to maintain a healthy body. For instance, our gut microbes metabolize dietary constituents and produce vitamins and amino acids our body cannot obtain by itself [7], while our oral microbes are responsible for dietary nitrate reduction thereby contributing to vasodilatation and other functions that are regulated by nitric oxide [8].

## 2. Oral Microbiome and Distant Pathologies

Since the advent of high-throughput technologies allowing microbial community assessment, the number of papers reporting on microbiome in relation to health is increasing nearly exponentially. Oral microbiome or specific oral taxa have been associated with extra-oral infections and inflammation [9] or even cancers [10,11]. Besides association, their involvement in the pathogenesis of distant tumors has been suggested. The proposed mechanisms involve interaction between a dysbiotic oral microbial community and the gastro-intestinal tract, via circulating microbial products in blood or via direct interaction of the gastro-intestinal tract tissue with individual oral microbes [12]. For instance, a study using quantitative PCR of *Fusobacterium nucleatum* in 99 colorectal carcinoma biopsies and 99 matched normal samples showed that colorectal carcinoma harbored on average 415 times higher counts of *F. nucleatum* than the healthy tissue [13]. *F. nucleatum* has been found in cyst fluid from pancreatic cysts that can progress to invasive pancreatic cancer [14], while both *F. nucleatum* and *Porphyromonas gingivalis* have been found in pancreatic cancer tissue samples [15].

Two prospective cohort studies can be seen as milestones in the assessment of oral microbiome–pancreatic cancer relation. A study by Michaud and colleagues in 2013 assessed antibodies to oral bacteria in pre-diagnosis blood samples of 405 pancreatic cancer cases and 416 matched controls and found a two-fold higher risk of pancreatic cancer in individuals with high levels of antibodies against *P. gingivalis* [16]. A study by Fan and colleagues in 2018 compared 361 pancreatic adenocarcinoma cases and 371 matched controls with oral wash samples collected 2–10 years before cancer diagnosis and found that oral carriage of *P. gingivalis* and *Aggregatibacter actinomycetemcomitans* was associated with 1.5- and 1.6-times higher risk of developing pancreatic cancer, respectively [17].

In addition to the findings above, numerous other studies have led to increased enthusiasm and expectations that oral microbiome assessment can be used for diagnostic or prognostic purposes of such distant pathologies as gastro-intestinal tract cancers. This has resulted in cross-sectional case–control studies where oral microbiome, frequently collected as salivary or an oral rinse sample or via an oral mucosal swab, is used to predict the cancer cases or cancer severity [18,19,20,21]. For instance, 16 bacterial OTUs (operational taxonomic units) in oral mucosal swabs (several OTUs of *Prevotella, Anaerostipes, Porphyromonas, Neisseria, Haemophilus, Fusobacterium, Peptostreptococcus, Streptococcus, Alloprevotella, Megasphaera, Leptotrichia* and *Cardiobacterium*) were identified to classify colorectal carcinoma cases with the sensitivity of 53% and specificity of 96% [18]. When the same method was used with the microbiome of the fecal samples, the sensitivity of the model was only 22%, while the combination of the two samples increased the sensitivity for the detection of colorectal carcinoma cases to 76%. On the other hand, a multinational study involving saliva from pancreatic cancer cases and controls found a high false positive rate when a Japanese dataset was validated against a Spanish dataset or vice versa, or when the selected biomarker species were validated against saliva of cases and controls from other studies with diseases such as rheumatoid arthritis, colorectal carcinoma or diabetes mellitus [21]. Paradoxically, different studies identify the same species in oral samples associated with either increased or decreased risk for the same cancer type: e.g., genera *Leptotrichia* and *Streptococcus* have been associated with both decreased and increased pancreatic cancer risk [22].

## 3. Can Oral Microbiome Be Used to Predict a Distant Pathology?

Is there then a potential for oral microbiome to be used as a diagnostic or prognostic tool for a distant pathology? In order to answer this question, one needs to acknowledge the complexity of the oral ecosystem. The oral microbiome is acquired in the first years of life and is shaped by an interplay of various environmental, biological and behavioral factors [23,24]. Its composition follows different phases in one’s development and next to behavioral factors such as oral hygiene practices, sugar consumption or smoking, oral microbiome will vary by age, depending on different stages of dentition, but also by hormonal levels (e.g., during puberty, pregnancy, menopause), presence of intraoral appliances or dentures, and salivary properties [25,26]. Behavior in turn is related to one’s cultural, educational and economic situation. Therefore, it is impossible to provide a single, one-size-fits-all definition of a healthy oral microbiome. We have tried to define the boundaries of a healthy oral ecosystem in a rather homogeneous population—268 young and healthy Dutch adults, mostly university students—and found high heterogeneity in salivary microbiome among these individuals [27]. This heterogeneity was related to personal saliva properties such as pH and enzyme activities, but also gender and diet of the individual. In other words, even the individuals who had similar age and background and who were orally and systemically healthy, harbored different, highly personalized salivary bacterial communities. This exemplifies the difficulties one would be facing in dichotomizing populations into healthy and diseased based on their oral microbial composition.

To date, oral sample collection is largely only secondary to fecal samples in gastro-intestinal tract-centered studies and is performed without the involvement of oral health professionals, clinical oral diagnosis or gathering oral hygiene-related and oral health-related information [24]. Unlike organized actions for fecal sample standardization [28,29], no such attempts have been made for oral microbiome collection methods. Furthermore, our knowledge on the variance in oral microbiome explained by the specific factors that are known to influence microbial composition and their mutual interplay is still limited. Additionally, the methodology to target only bacteria (16S rRNA gene amplicon sequencing) has become a mainstream approach in microbiome research. This has led to very biased information on the oral microbiome as a whole: there is limited knowledge available on fungal communities (mycobiome), Archaea (archaeome), protozoa (parazitome) and viruses (virome)—the so called ‘dark matter’ of the oral microbiome [30,31,32].

## 4. Concluding Remarks

Taken together, we are currently scratching the tip of the iceberg regarding the full description of a healthy oral microbiome. This lack of knowledge implies that we have to be careful when using oral microbiome data in pinpointing deviations from this norm and relating these with prediction, diagnosis and prognosis of a (distant) pathology. Development of internationally accepted standard procedures for oral sample and metadata collection is urgently needed to unravel the full potential of the insights that might be obtained from the assessment of oral microbiota in relation to general health.

## Data Availability

This study did not report any data.
